# Fossil evidence and stages of elongation of the *Giraffa camelopardalis* neck

**DOI:** 10.1098/rsos.150393

**Published:** 2015-10-07

**Authors:** Melinda Danowitz, Aleksandr Vasilyev, Victoria Kortlandt, Nikos Solounias

**Affiliations:** 1 Department of Anatomy, New York Institute of Technology College of Osteopathic Medicine, Old Westbury, NY 11568-8000, USA; 2 Department of Biomedical Sciences, New York Institute of Technology College of Osteopathic Medicine, Old Westbury, NY 11568-8000, USA

**Keywords:** neck, elongation, Giraffidae, *Samotherium*, cervical vertebrae

## Abstract

Several evolutionary theories have been proposed to explain the adaptation of the long giraffe neck; however, few studies examine the fossil cervical vertebrae. We incorporate extinct giraffids, and the okapi and giraffe cervical vertebral specimens in a comprehensive analysis of the anatomy and elongation of the neck. We establish and evaluate 20 character states that relate to general, cranial and caudal vertebral lengthening, and calculate a length-to-width ratio to measure the relative slenderness of the vertebrae. Our sample includes cervical vertebrae (*n*=71) of 11 taxa representing all seven subfamilies. We also perform a computational comparison of the C3 of *Samotherium* and *Giraffa camelopardalis*, which demonstrates that cervical elongation occurs disproportionately along the cranial–caudal vertebral axis. Using the morphological characters and calculated ratios, we propose stages in cervical lengthening, which are supported by the mathematical transformations using fossil and extant specimens. We find that cervical elongation is anisometric and unexpectedly precedes Giraffidae. Within the family, cranial vertebral elongation is the first lengthening stage observed followed by caudal vertebral elongation, which accounts for the extremely long neck of the giraffe.

## 1. Introduction

The evolution of the elongated giraffe neck has been a topic of interest to scientists since evolution was theorized by Lamarck and Darwin. It has been popularly demonstrated that this adaptation permits giraffes to use vegetation at higher levels from the ground, therefore outcompeting the shorter-necked herbivores, and allows for a specialized mode of fighting for male dominance in mating, termed ‘necking’ [1,2]. Analysis of rate of neck mass increase found little difference between male and female giraffes, suggesting a smaller role of sexual selection in the elongation of the neck [3]. It has been proposed that both foraging and male combat probably contributed to giraffe neck lengthening; observation of giraffes browsing from tree heights up to 5 m favours the ‘competing browsers’ hypothesis, and the direct selection of larger-necked males by oestrous females supports the sexual selection theory [4].

Until now, the study of this remarkable evolutionary feat has been largely limited to analysis of the extant giraffe neck. The physiological mechanisms regulating blood pressure and cerebral perfusion to compensate for the massive increase in neck length have been extensively studied [5–7]. Several studies have addressed the unique specializations of the *Giraffa camelopardalis* C7 and T1 vertebrae, suggesting atypical cervicothoracic anatomic features, possibly contributing to neck lengthening [8,9]. An osteological study of foetal and adult giraffe vertebrae concluded that substantial cervical lengthening occurs after birth [10]. While the extant giraffe neck has been adequately researched, osteological demonstration of the fossils and evolutionary transformation of the neck is lacking. One study focusing on the vertebral lengths of extant ungulates and extinct giraffids found that giraffid cervical elongation was present among the palaeotragines (approx. 12 Ma) [9]. Examination of fossil cervical vertebrae is necessary for a comprehensive understanding of the evolution of the giraffe neck, and how the cervicals lengthen. We provide and evaluate measurements and describe detailed anatomic characteristics on cervical vertebrae of extant and extinct giraffids representing all seven subfamilies to relate the phylogeny to the evolution of neck elongation. We use these morphological features, as well as a mathematic vertebral transformation, to determine regions of the cervicals exhibiting significant lengthening. The extinct taxa in question are not direct ancestors to the giraffe, yet their neck structure provides intermediate stages culminating in the extraordinary elongation of the *G. camelopardalis* neck.

## 2. Methods

**Figure 1.**
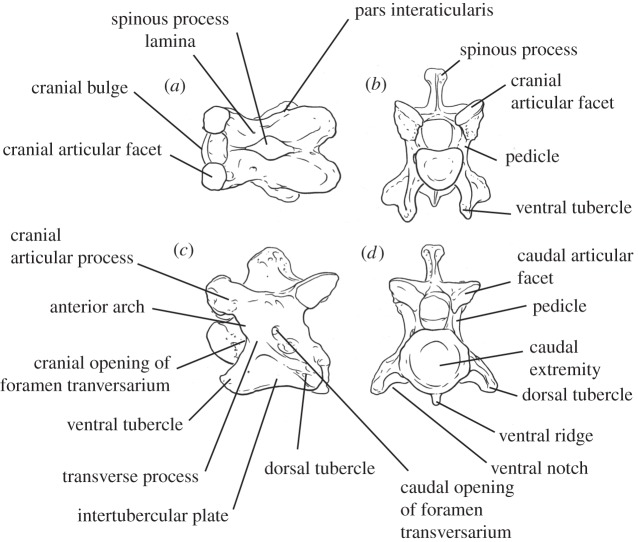
Labelled C3 *Okapia johnstoni* (AMNH 51215) vertebra depicting terminology used for describing cervical anatomy as well as for elongation characters. (*a*) Dorsal view, (*b*) cranial view, (*c*) lateral view, and (*d*) caudal view.

**Figure 2.**
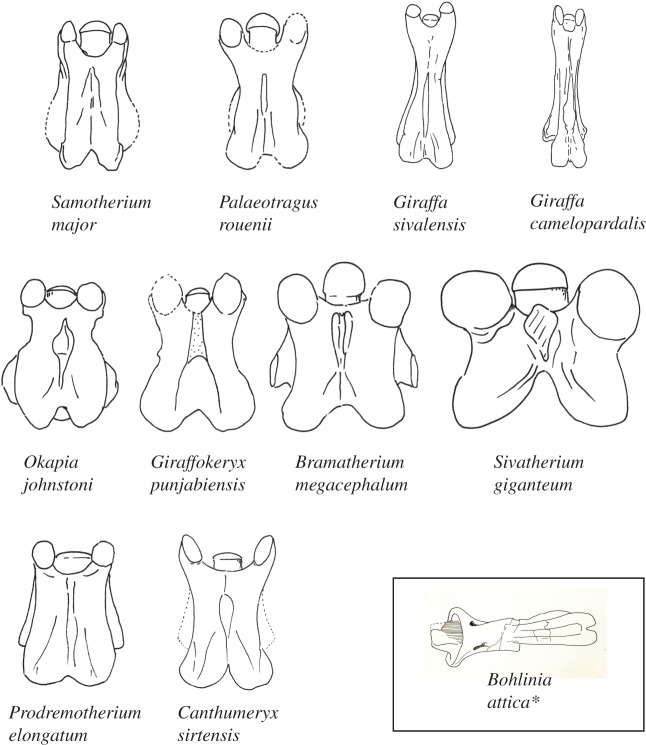
Demonstration of a dorsal view of a representative C3 vertebra for all giraffids evaluated in this study. Each specimen is isometrically scaled so that all specimens are of equal length. The most primitive giraffid (*Canthumeryx sirtensis*) and sister taxon (*Prodremotherium elongatum*) comprise the bottom row; the giraffids with secondarily shortened C3 vertebrae (*Okapia johnstoni, Giraffokeryx punjabiensis, Sivatherium giganteum* and *Bramatherium megacephalum*) comprise the middle row; the giraffids with progressive vertebral elongation comprise the top row (*Samotherium major, Palaeotragus rouenii, Giraffa sivalensis* and *Giraffa camelopardalis*). *As there are no known C3 vertebrae of *Bohlinia attica*, a representative C2 of this taxon is illustrated in the black square.

**Figure 3.**
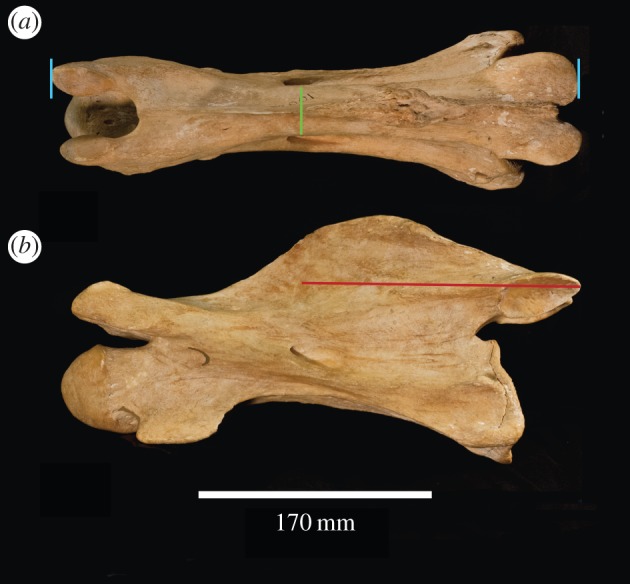
Schematic of measurements used to quantify cervical vertebral elongation. *Giraffa camelopardalis* C3 (AMNH 82001) in (*a*) dorsal and (*b*) lateral views. The light blue lines represent maximum length (distance between the cranial and caudal articular facets. The green line represents minimal width. The red line represents the portion of the vertebra caudal to the caudal opening of the foramen transversarium.

Using morphological features of 71 giraffid cervical vertebrae comprising 11 species, we developed 20 character states to evaluate and compare elongation among species (electronic supplementary material, table S1). Measurements and character evaluation were done using callipers in millimetres, from high-quality specimen photographs. These characters qualify features of the spinous process, lamina, cranial and caudal articular facets, cranial bulge of the vertebral body, ventral and dorsal tubercles, and foramen transversarium (figure 1 and see the electronic supplementary material for more information about the characters). We have selected the C3 vertebra to be used for the majority of characters as a representative of vertebral morphology and total neck elongation. The length of cervical vertebra tends to slightly decrease progressively towards the thorax and C3–C6 vertebrae are relatively similar in length [9]. We therefore chose the C3 vertebra to evaluate and compare lengthening between giraffid species (figure 2). We also evaluate several characters on C2 to incorporate *Bohlinia*, a close ancestor to *Giraffa*, into our study, and to achieve a more comprehensive analysis of cervical elongation within Giraffidae. Ten characters relate to general vertebral elongation, five relate to elongation of the cranial portion of the vertebra and six characters relate to elongation of the caudal portion of the vertebra. We provide working hypotheses to show how our character states relate to vertebral lengthening. The slenderness of the vertebra, a key feature of elongation, is measured by calculating the ratio between the maximal length of the cervical vertebra and the minimal width of the lamina in dorsal view (a ratio closer to 1 reflects a squared vertebra, indicating that it is proportionately short). The percentage of caudal vertebral length is calculated by measuring the length of the entire vertebra (distance between cranial and caudal articular facets), and the length of the vertebra caudal to the foramen transversarium (distance between caudal opening of the foramen and the most caudal part of the caudal articular facet), and dividing the total length by the caudal length (figure 3). All measurements were taken by N.S.

We use minimal width of the dorsal lamina and maximum vertebral length as measurements to evaluate elongation because these are convenient and easily reproducible calculations using standard specimen photographs (electronic supplementary material). In giraffids, the structures around the vertebral body are often curved from cranial to caudal, and the resulting centrum length is therefore an inadequate measurement, as it underestimates the length. The dorsal lamina, however, is a flat surface, providing a more accurate measure of vertebral length. In addition, the maximum length encompasses lengthening of the cranial and caudal articular processes, which are important aspects of vertebral elongation in ungulates. Lastly, our measurements are designed so they can be easily performed on articulated necks, where the caudal vertebral body cannot be easily measured. For these reasons, we do not apply the standard elongation index to the giraffid cervicals [11,12].

By examining articulated necks of *Sivatherium, Bramatherium, Okapia* and *G. camelopardalis*, and relatively complete necks of *Samotherium*, we found morphological features to distinguish C3 from C4 to C5. Notably, the spinous process of all examined giraffids becomes longer, and the width of the base of the spinous process becomes more confined from C3 to C5. Using these features, we believe that the C3 vertebrae in our sample are correctly identified. However, if any C4–C5 specimens were mistakenly identified as C3, this would have a minor effect on our results, as the majority of the characters, in addition to vertebral length and minimal width, are relatively consistent from C3 to C5.

**Figure 4.**
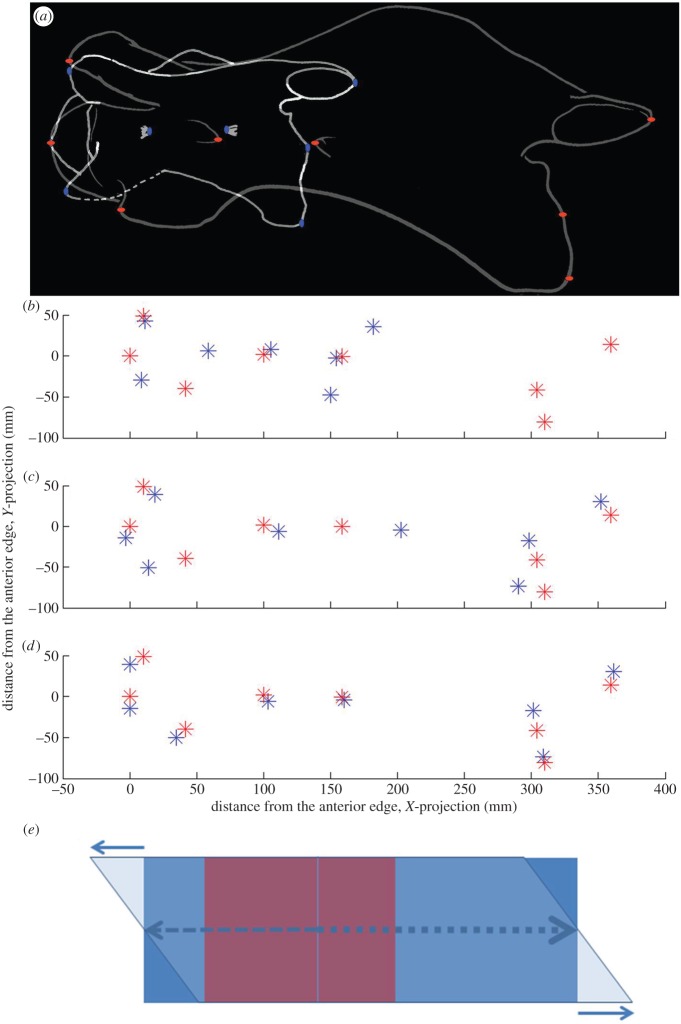
Computational model accounts for the third cervical vertebra change between *Samotherium* (blue) and modern giraffe (red). (*a*) Aligned C3 of *Samotherium* and *G. camelopardalis* show marker placement corresponding to the cranial-most point of the cranial articular facet, caudal-most point of the caudal articular facet, cranial-most point of the cranial bulge, cranial and caudal openings of the foramen transversarium, dorsal-most point of the caudal vertebral body, ventral-most point of the caudal vertebral body, and cranial-most tip of the ventral tubercle. (*b*) Eight marker positions in aligned lateral views of *Samotherium* and *G. camelopardalis* C3. (*c*) The best fit of a uniform linear ‘stretch’ model applied to the *Samotherium* fitted to the modern giraffe marker position. The best-fit uniform linear ‘stretch’ coefficient is 2.0 (95% confidence interval (CI) =1.7–2.3). (*d*) The best fit of stretch–slide model applied to the *Samotherium* against the modern giraffe marker position. The model predicts that the transition from a modest to marked stretch occurs caudal to the posterior opening of the foramen transversarium 128 mm from the cranial aspect of the bone (95% CI= 92–166 mm) and the corresponding ‘stretch’ coefficients are 1.8 (95% CI= 1.5–2.1) cranial to that point and 2.9 (95% CI=2.5–3.2) caudal to that point (*p*-value for comparing cranial and caudal elongation coefficients was =0.003). In addition, the model required a 38 mm ‘sliding’ of dorsal with relationship to the ventral vertebra (95% CI=20–58 mm). If no sliding is included in the model, the best predicted values for the transition point and the cranial and caudal ‘stretch’ coefficients are: 122 mm (95% CI=18–226 mm), 1.7 (95% CI=1.0–2.5) and 2.7 (95% CI=1.9–3.5). (*e*) A schematic of a transformation used in the model. Red rectangle represents the original object. The blue rectangle represents the stretched object, where stretching to the right (caudal) of the vertical line is greater than that to the left of the line. Superposition of horizontal sliding results in the final shape, represented here by the parallelogram.

We also perform mathematical transformation of a third cervical vertebra of *Samotherium*, a key extinct giraffid, to the proportions of *G. camelopardalis* to analyse the loci where the greatest cervical lengthening occurs. The *Samotherium* specimen was ideal for analysis as it is well preserved, and its location on the cladogram marks the transition to the longer-necked giraffids. Lateral views of the *Samotherium* and *G. camelopardalis* C3 vertebrae were scaled to the same size. Eight markers on the vertebrae were used, and relative marker positions were determined using ImageJ. All the positions were measured relative to the cranial-most point, which corresponds to the cranial tip of the cranial bulge. Custom linear model fits were performed using Matlab curve-fitting toolbox and Levenberg–Marquardt least-squares regression algorithm. Horizontal or vertical coordinates of the eight markers in the *Samotherium* C3 vertebral sample were used as input variables, whereas the corresponding measurements in the giraffe C3 vertebra were used to test model output. The following models were used: (i) uniform ‘stretch’ model: *y*=a*x+*x*0, (ii) non-uniform ‘stretch’ model: *y*=(*a*1, if *x*<*x*0 or *a*2, if *x*>*x*0)*x+*x*0, and (iii) ‘stretch and slide’ model: *y*=(*a*1, if *x*<*x*0 or *a*2, if *x*>*x*0)*x+*x*0+b*dx (where *b*=1 for ventral markers: ventral-most point of the caudal vertebral body, and cranial-most tip of the ventral tubercle, *b*=0 for midline markers: cranial-most point of the cranial bulge, cranial and caudal openings of the foramen transversarium, and dorsal-most point of the caudal vertebral body; *b*=−1 for dorsal markers: cranial-most point of the cranial articular facet, and caudal-most point of the caudal articular facet). Introduction of the b*dx component was necessary to account for an apparent cranial ‘sliding’ of the dorsal aspect of the modern giraffe bone relative to ventral when compared with *Samotherium*. The results were plotted in Matlab and assembled in Photoshop (figure 4).

**Figure 5.**
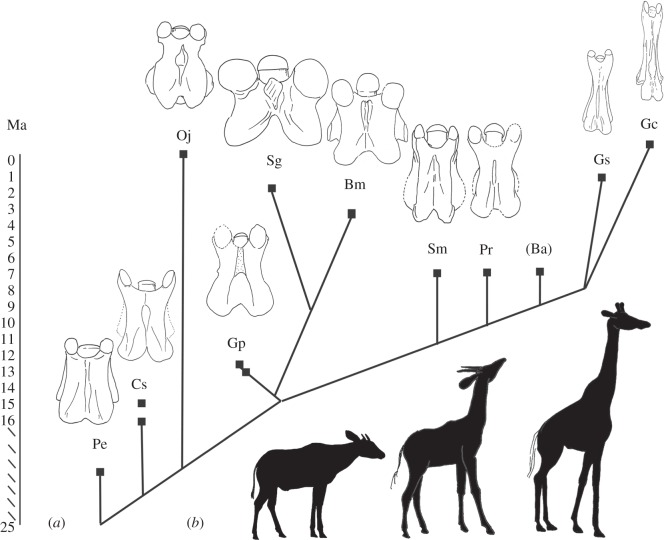
Cladogram with geological age and dorsal view of C3 vertebrae of taxa evaluated. Pe, *Prodremotherium elongatum*; Cs, *Canthumeryx sirtensis*; Oj, *Okapia johnstoni*; Gp, *Giraffokeryx punjabiensis*; Sg, *Sivatherium giganteum*; Bm, *Bramatherium megacephalum*; Sm, *Samotherium major*; Pr, *Palaeotragus rouenii*; Ba, *Bohlinia attica*; Gs, *Giraffa sivalensis*; Gc, *Giraffa camelopardalis*. (*a*) A modified cladogram based on previously published cladograms by Hamilton [13] and Solounias [14], with the exclusion of species not evaluated in this study. Each clade terminates in a square point corresponding to the age of the respective taxon in millions of years (Ma). The dorsal view of a C3 vertebra for each taxon is demonstrated (excluding *Bohlinia* as there are no known C3 fossils). Each specimen is isometrically scaled so that all vertebrae are of equal length. (*b*) Silhouettes of *O. johnstoni, S. major* and *G. camelopardalis* (left to right) are provided to give a comprehensive image of a long-necked, short-necked and intermediate-necked individual.

There are few giraffid fossil cervical vertebrae, which are scattered throughout museums worldwide. We used almost every identifiable C2, C3 and C7 specimen known for this study (electronic supplementary material, table S2). Many of these fossils are fragmented and/or plastically deformed, therefore, in certain instances, it was necessary to compile characters from multiple specimens. Character states were confirmed with all specimens available for each species analysed. The species studied come from all seven giraffid subfamilies, therefore, our sample is comprehensive of Giraffidae. The age span is from the onset of the family (approx. 19–16 Ma) to the extant species (figure 5).

## 3. Results

### 3.1 Evaluation of character states and C3 length-to-width ratio

Character analysis and length-to-width ratio calculations demonstrate that *Prodremotherium* and *Canthumeryx* exhibit intermediate cervical lengthening. These taxa demonstrate several general elongation characters, including low, thin and horizontal spinous processes, and a shallow concave pars interarticularis (electronic supplementary material, table S1). Specimens of both *Prodremotherium* and *Canthumeryx* have a C3 length-to-width ratio suggesting moderate elongation had already occurred (*Prodremotherium* range: 1.8–2.11, *Canthumeryx* range: 3.16–3.36); these vertebrae are twice to three times as long as they are wide (electronic supplementary material, table S3).

There are several taxa with secondarily shortened cervical vertebrae compared with *Canthumeryx*. All subsequent giraffids possess necks progressively further elongated from an already lengthened state (figure 5).

*Okapia, Giraffokeryx, Sivatherium* and *Bramatherium* have C3 length-to-width ratios that are short, with *Sivatherium* exhibiting the shortest C3 of all giraffids (*Okapia* range: 1.41–2.07, *Giraffokeryx* range: 2–2.21, *Sivatherium*: 1.21, *Bramatherium*: 1.3). Unlike the other elongated-neck giraffids, in these taxa, the C2 spinous process is high, thick and inclined vertically, and in all except *Bramatherium*, the C2 pars interarticularis is deep-concave shaped, character states consistent with the non-elongate vertebrae (figure 1 and electronic supplementary material, table S1).

**Figure 6.**
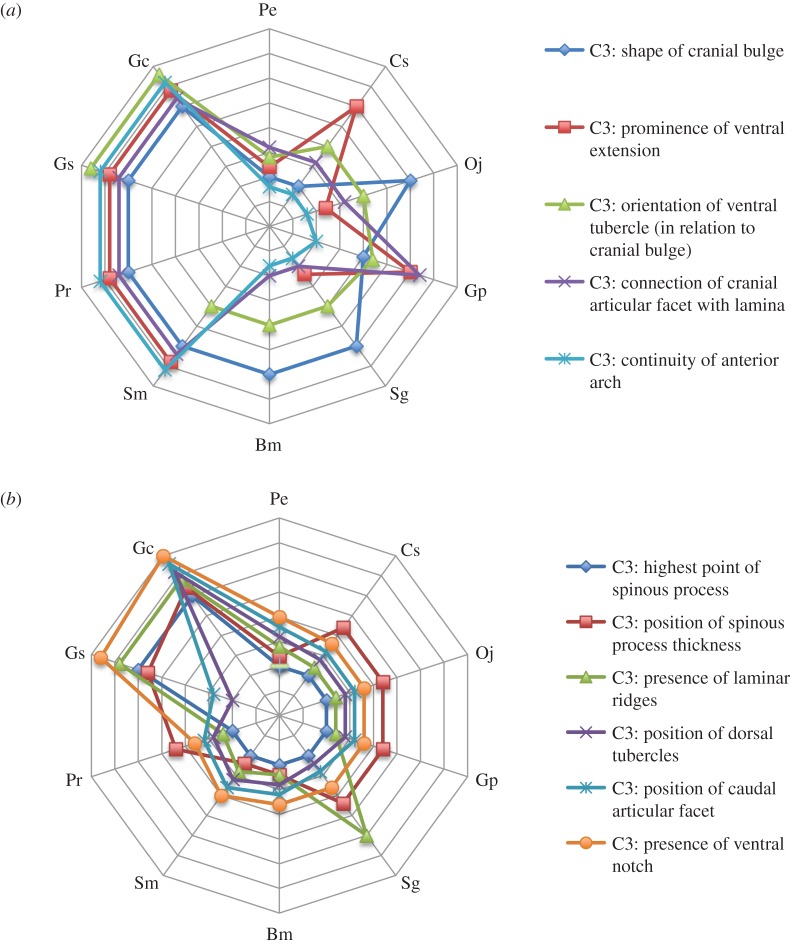
Radar plot shows cranial and caudal elongation of giraffid C3 vertebrae. Pe, *Prodremotherium elongatum*; Cs, *Canthumeryx sirtensis*; Oj, *Okapia johnstoni*; Gp, *Giraffokeryx punjabiensis*; Sg, *Sivatherium giganteum*; Bm, *Bramatherium megacephalum*; Sm, *Samotherium major*; Pr, *Palaeotragus rouenii*; Ba, *Bohlinia attica*; Gs, *Giraffa sivalensis*; Gc, *Giraffa camelopardalis.* Y-axes represent giraffid species. *X*-axes demonstrate the degree of elongation of a given character; the centre of the plot represents the shortened state while the lateral portion of the plot represents the elongation state. Each star plot represents a specific vertebral characteristic. (*a*) Radar plot depicting the cranial elongation characters. *S. major*, *P. rouenii*, *G. sivalensis* and *G. camelopardalis* exhibit the greatest degree of cranial elongation. (*b*) Radar plot depicting the caudal elongation characters. A caudal pull on C3 can be seen in *G. sivalensis*, and is most pronounced in *G. camelopardalis*.

The remaining taxa (*Samotherium*, *Palaeotragus*, *Giraffa sivalensis* and *G. camelopardalis*) display the elongation state for characters qualifying cranial vertebral elongation of C3 (figure 6*a*). There are no known C3 vertebrae of *Bohlinia*; therefore, cranial elongation cannot be evaluated. Notably, these taxa share the presence of a prominent ventral extension, a disconnected cranial articular facet from the dorsal lamina, and an interrupted anterior arch (electronic supplementary material, table S1). The C3 length-to-width ratios for *Samotherium* and *Palaeotragus* indicate moderate cervical lengthening (*Samotherium* range: 2.26–3.75, *Palaeotragus* range: 2.87–3.72).

*Giraffa sivalensis* possesses the most elongated C3 vertebra of the extinct giraffids. This C3 vertebra is the most similarly proportioned to the C3 of the extant giraffe, as evidenced by the slender length-to-width ratio (6.67). This taxon shares all cranial and most general lengthening features of the elongated giraffids, as well as several caudal elongation character states, shared only with *G. camelopardalis* (figure 6*b*). Namely, the highest point of the spinous process is located caudal to the foramen transversarium, there are no laminar ridges and the ventral notch is absent in both *Giraffa* species (electronic supplementary material, table S1).

The modern giraffe exhibits several character states indicative of caudal cervical elongation unique to all other giraffids (figure 6*b*). In addition, this taxon exhibits all general and cranial elongation character states with the exception of one, and has the largest C3 length-to-width ratio of all taxa evaluated (ratio: 8.88–10.8). Its dorsal tubercles, which protrude in all other giraffids, are intimately attached to the centrum, and the caudal articular facet is displaced caudal to the vertebral body (electronic supplementary material, table S1). The caudal portion of C3 (caudal to the foramen transversarium) is the largest portion of the vertebra, comprising an impressive 50.7–56.3% of total vertebral length, which is greater than that of all other giraffids (electronic supplementary material, table S3).

### 3.2 Computational comparison of C3 of *Samotherium* with *Giraffa camelopardalis*

The simplest hypothesis about the transformation from *Samotherium* to *G. camelopardalis* is that the vertebra gets longer in the cranial–caudal direction. However, linear regression showed that it is impossible to obtain accurate matching of the corresponding landmarks using this simple model (figure 4*c*). We therefore propose that there exists a transition point that determines the degree of elongation (with different coefficients cranial and caudal to that point). We also propose an additional sliding of the dorsal aspect of the vertebra relative to its ventral side (as shown in figure 4*e*). The resulting linear regression is significantly more accurate (figure 4*d*) and predicts that the cranial and caudal ‘stretch’ coefficients are indeed significantly different (1.8±0.15 and 2.9±0.2, *p*=0.003). Independently determined vertical stretching shows that the predicted ‘stretch’ coefficient is very slight and not statistically different from one (1.2±0.2).

## 4. Discussion

We examine neck elongation throughout giraffid evolution by plotting the 20 character states against previously published cladograms [13,14]. This allows for the observation of patterns of vertebral lengthening throughout time, and for the analysis of giraffid evolutionary relationships solely using cervical vertebral morphological features.

### 4.1 The subfamilies of Giraffidae

#### 4.1.1 Canthumerycinae

Hamilton [13] erected a new family for *Canthumeryx* (Canthumerycidae). Solounias [14] has integrated *Canthumeryx* within Giraffidae as the subfamily Canthumerycinae. These giraffids have primitive dentition, an open lasolacrimal canal and a small secondary lobe on the canine. This includes *Georgiomeryx georgalasi* and *Canthumeryx sirtensis*. Material is known from Muruarot Hill of Kenya (17 Ma), Gebel Zelten of Libya (18–16 Ma) and Fort Ternan of Kenya (14 Ma).

#### 4.1.2 Okapiinae

This is represented by *Okapia johnstoni* and *Afrikanokeryx leakey* [15]. *Okapia johnstoni* is one of two extant members of Giraffidae. The extant animal is known from Zaire, and fossils are known from Ngorora of Kenya (9 Ma).

#### 4.1.3 Giraffokerycinae

These giraffids are characterized by the presence of long metapodials, four ossicones and the absence of frontal sinuses [14]. This includes *Giraffokeryx punjabiensis* and *Giraffokeryx primaevus*. Material is known from Fort Ternan of Kenya (14 Ma) and the Chinji Formation of Pakistan (14 Ma).

#### 4.1.4 Sivatheriinae

Bonaparte 1850. These giraffids have four ossicones, large frontal sinuses and a large body size compared with other giraffids and coexisting ungulates. This includes the species of *Bramatherium, Sivatherium, Birgerbohlinia* and *Helladotherium*. Material is primarily known from the Pliocene Siwaliks (5–2 Ma), Pikermi of Greece (approx. 7 Ma), Samos of Greece (7.5 Ma), Piera of Spain and Langebaanweg of South Africa (2 Ma) [13].

#### 4.1.5 Palaeotraginae

Pilgrim [16]. Hou *et al*. [17] included Samotheriinae in this subfamily. These giraffids have bare ossicones with wear facets, and small frontal sinuses. This includes the species of *Schansitherium, Samotherium, Palaeotragus* and *Alcicephalus*. Material is primarily known from Samos of Greece (7.5 Ma), Pikermi of Greece (approx. 7 Ma), Taskinpasa, Kemiklitepe and Sinap of Turkey and Maragheh of Iran (approx. 7.5 Ma) [18].

#### 4.1.6 Bohlininae

These giraffids have long and slender metapodials with a deep trough, and the absence of cranial sinuses [14]. This includes *Bohlinia attica* and *Honanotherium schlosseri*. Material is primarily known from Gansu and Shanxi of China, Pikermi of Greece (approx. 7 Ma) and Samos of Greece (7.5 Ma).

#### 4.1.7 Giraffinae

Zittel 1893. These giraffids have the longest metapodials with no trough, and large sinuses. This includes *Giraffa camelopardalis, Giraffa sivalensis, Giraffa jumae, Giraffa pygmaea, Giraffa stillei* and *Giraffa gracilis.* The extant animal is found throughout Africa, and fossils are known primarily from the Upper Siwaliks of Pakistan (approx. 2–3 Ma), Koobi Fora and Rawi of Kenya, and Olduvai of Tanzania (2 Ma) [19].

### 4.2 Hypotheses relating character states to vertebral elongation

Our 20 character states evaluate elongation of various aspects of the cervical vertebrae using anatomic features (figure 1). The height and transverse thickness of the spinous process relates to general vertebral elongation; vertebral body-lengthening gaps the distance between the occipital bone and cranial portion of the spinous process, which thins the bony material comprising the spinous process. The base of the spinous process attaches to the dorsal lamina, and as both the lamina and the base are stretched with vertebral lengthening, we believe the spinous process is pulled down, resulting in a shorter height. The shape of the pars interarticularis and height of the intertubercular plate relate to the distance between the cranial and caudal articular facets, and ventral and dorsal tubercles, respectively. Elongation increases the distance between these bony protrusions, resulting in a shallow–concave pars interarticularis and excavated intertubercular plate.

Characters evaluating anatomic features of the cranial portion of the vertebra relate to cranial vertebral elongation. The anterior arch is the ridged connection between the cranial articular process and ventral tubercle. This arch is interrupted during cranial elongation; the vertebral body lengthens cranial to the arch and displaces the base of the cranial articular process caudally. Oval-shaped cranial articular facets are also congruent with cranial vertebral elongation. This occurs owing to lengthening of the cranial articular process, onto which the facet attaches. The shape change of the cranial facet probably accompanies a parallel change of the caudal facet of the preceding vertebra.

Caudal vertebral lengthening is qualified by character states relating to the caudal portion of the C3 vertebra. One consequence of caudal elongation is the displacement of the thickest portion and highest point of the spinous process caudal to the foramen transversarium. In addition, we believe caudal projection of the vertebral body fills the ventral notch, located on the caudal aspect of the lamina, with bony material.

### 4.3 Elongation precedes Giraffidae

*Prodremotherium* is considered to be a potential ancestor of Giraffidae based on fused and elongated metapodials, small canines and reduced cingulum on upper molars [20]. *Canthumeryx* is a basal giraffid with its open nasolacrimal canal and protruding occipital [13]. Examination of *Prodremotherium* and *Canthumeryx* specimens reveals that not only did the giraffid lineage begin with a relatively elongated neck, but that this cervical lengthening precedes Giraffidae (figure 5). Surprisingly, there are few cervical features that differentiate *Prodremotherium*, a potential giraffid ancestor, from *Canthumeryx*, the most primitive giraffid, as both these animals have necks in an intermediate stage of elongation. Evidently, the features uniting Giraffidae are the presence of ossicones, a bilobed canine and a larger body size [14]. It is novel that neck length, the most distinguishing and popular attribute of *Giraffa*, is apparently not a defining feature of the family.

### 4.4 Secondary shortening of cervical vertebrae

*Okapia, Giraffokeryx, Sivatherium* and *Bramatherium* have C3 length-to-width ratios secondarily shortened compared with *Canthumeryx.* The two sivatheres (*Sivatherium* and *Bramatherium*) possess exceptionally robust and massive C3 vertebrae, consistent with their large body size and broad metapodials (figure 2) [14]. These four taxa are the only giraffids with a thick C2 spinous process, and they exhibit the elongation state of very few characters. It is possible that these taxa are united by a common ancestor, and that secondary shortening occurs once in the phylogeny. It is also feasible that this shortening occurs several times convergently. The secondary shortening appears to have occurred relatively recently, as *Okapia*, *Sivatherium* and *Bramatherium* are geologically more recent, spanning 3 Ma to the present (figure 5). *Giraffokeryx* is an older taxon, however, its length-to-width ratio and number of elongation character states indicates that the vertebrae are more elongated than the other three non-elongate giraffids. It has been suggested that *Giraffokeryx* is a plausible ancestor for the sivatheres [14].

### 4.5 Cranial vertebral elongation

We believe elongation of the cranial portion of the vertebrae accounts for the moderate elongation exhibited by *Samotherium* and *Palaeotragus*, and contributes to the substantial elongation seen in both *Giraffa* species (figure 6*a*). These taxa all share the presence of a prominent ventral extension, which describes a caudal lengthening of the caudal portion of the cranial bulge. They also possess a disconnected cranial articular facet from the lamina; the cranial articular facet connects to the lamina via the cranial articular process, which lengthens and displaces the facet cranially during cranial vertebral elongation. While the cranial articular process is present in all giraffids, in the non-elongate state, the process is too short to fully disconnect the cranial facet from the dorsal lamina. We believe cranial lengthening is the first stage of cervical elongation seen in Giraffidae. This is consistent with an analysis of giraffid vertebral lengths, where it was found that cervical lengthening occurred among the palaeotragines [9]. This is a pivotal point in giraffid neck evolution.

### 4.6 Caudal vertebral elongation

Giraffidae began with a partially elongated neck, which was further stretched by cranial vertebral lengthening approximately 7.5 Ma (figure 5). The final stage, which resulted in the long neck of *G. camelopardalis*, is caudal vertebral elongation. *Giraffa sivalensis* appears to be transitional, as it shares a few but not all caudal elongation character states with *G. camelopardalis* (figure 6*b*). One consequence of caudal elongation is the displacement of the thickest portion of the spinous process caudally. The bony concentration is central and appears to be expanding caudally in *G. sivalensis*, whereas the material is completely caudal in *G. camelopardalis.* Presumably, this caudal lengthening is the final phase culminating in the extremely long neck of the giraffe. Correspondingly, the caudal portion of the C3 of *G. camelopardalis* comprises the majority of total vertebral length. In agreement with our findings, the vertebral body measurements of *Giraffa* sp. from Koobi Fora scaled similarly to those of the lengthened *G. camelopardalis* cervicals [9]. Reconstructions of the body size of *G. sivalensis* suggest that it was scaled similarly to that of *G. camelopardalis* [21]. Caudal elongation, combined with the earlier cranial elongation from an already lengthened state, ultimately leads to the remarkable *Giraffa* neck.

### 4.7 Analysis of *Bohlinia* based on the axis and C7

*Bohlinia* is difficult to analyse as C3, the principal vertebra evaluated for elongation, is lacking. However, every character state observed on the axis, of which there are three specimens, exhibits significant lengthening. The spinous process is low, thin and oriented horizontal to the vertebral body, all characteristics of an elongated vertebra (electronic supplementary material, table S1). We can also extrapolate that this taxon had a long neck based on its tall metapodials; measurements reveal that the metapodials were similarly proportioned and sized as those of *G. camelopardalis* [22]. The combination of a short *Bohlinia* neck with elongated limbs is not feasible, as it would prevent the animal from attaining water at ground level. Two hypotheses exist relating neck and limb lengthening; one proposes that limbs and necks elongate at the same pace, and the second suggests that cervical vertebrae elongate disproportionately faster than the metapodials [4]. Neither scenario allows for limb elongation to precede that of the neck. Lastly, measurements of the C2 and C7 of *Bohlinia* specimens demonstrate that these vertebrae are elongated. The *Bohlinia* C2 length-to-width ratio ranges from 5.52 to 5.85, a value second only to *G. camelopardalis*. Likewise, the C7 length-to-width ratio ranges from 1.88 to 2.14, greater than all other giraffids with the exception of the modern giraffe (electronic supplementary material, table S3). The elongation and slenderness of both the proximal and distal vertebrae lead us to infer that the middle cervical vertebrae are also lengthened.

### 4.8 The unique C7 of *Giraffa camelopardalis*

*Giraffa camelopardalis* has a C7 with several specializations, unique not only to giraffids, but also to mammals. The giraffe C7 possesses the dorsal and ventral tubercles, and has flat and continuous transverse process, features consistent with more proximal cervical vertebrae [23,24]. Notably, the vertebral artery enters the foramen transversarium of C7 in *G. camelopardalis*, whereas it enters at C6 in other mammals [9]. In addition, the giraffe T1 has been proposed as ‘cervicalized’, supported by the location of the brachial plexus and its articulations with rib 1, which on other mammals occur on C7 [8]. *Giraffa camelopardalis* is also unique in that it has an elongated C7. The length-to-width ratio of C7 (range: 3–3.75) is greater than that of any other giraffid or ancestor. The length of C7 is typically more conservative among mammals, exemplified by the camel and llama where the neck is elongated, but C7 remains shorter [25]. The cervicothoracic junction is pivotal for movement of the neck [8]. We believe these unique specializations on C7 and T1 allow for this elongation of C7 seen only in *G. camelopardalis*, further contributing to neck elongation.

## 5. Conclusion

The examination and evaluation of giraffid fossil specimens not only provide evidence for the evolution of cervical elongation, but also demonstrate the loci where lengthening occurred. Using morphological features and length/width measurements of C2 and C3 vertebrae, as well as computational comparisons of fossil with extant vertebral specimens, we provide a comprehensive analysis of cervical elongation. We propose that cervical elongation precedes Giraffidae. We also believe that cranial lengthening is the first stage of elongation seen in the family, followed by caudal lengthening, which accounts for the extreme *Giraffa* neck elongation. Mathematical transformation further supports the proposed stages, where both cranial and caudal elongation contribute to the illustrious *G. camelopardalis* neck.

## Supplementary Material

The supplementary text includes descriptions of the characters used to evaluate elongation of the vertebrae. It also includes a table of the characters and the character state exhibited by each giraffid species, a table of specimens with museum numbers, and a table of ratios and percent of the vertebrae caudal to the foramen transversarium.

## Supplementary Material

We are also providing a supplemental .excel file with all measurements.

## Data accessibility

The measurements supporting this article have been uploaded in the electronic supplementary material.
