# Supplementary material for: Fossil evidence and stages of elongation of the Giraffa camelopardalis neck
Source: R Soc Open Sci. 2015 Oct 7;2(10):150393. doi: 10.1098/rsos.150393 (PMC4632521; doi:10.1098/rsos.150393)
Supplement: The supplementary text includes descriptions of the characters used to evaluate elongation of the vertebrae. It also includes a table of the characters and the character state exhibited by each giraffid species, a table of specimens with museum numbers, and a table of ratios and percent of the verte [file rsos150393supp1.docx]

**Description of Characters**

Note: parenthesis indicates character state indicative of elongation

Height of spinous process C2: (low indicates elongation) Compares the height of the spinous process to the depth of the vertebral body. If the height of the spinous process is equal to or less than the depth of the vertebral body, we term this “low,” and if the height is greater than the depth of the vertebral body we term this “high.”

Height of spinous process C3: (low indicates elongation) Compares the height of the spinous process to the depth of the vertebral body. If the height of the spinous process is ½ or less the depth of the vertebral body, we term this “low” and if the height is greater than ½ the depth of the vertebral body, we term this “high.” We use different calculations to compare the height of the spinous process of the axis and C3 because the inherent structure of these vertebrae is different; the spinous process of the axis is consistently taller in most animals.

Overall thickness spinous process C2: (thin indicates elongation) Compares the width of the shaft of the spinous process to that of the lamina in dorsal view. We describe this as thick when greater than 50% of the shaft of the spinous process has a width greater than the width of the lamina. We call this thin when less than 50% of the spinous process has a width greater than that of the lamina.

Verticality of spinous process C2: (horizontal indicates elongation) Creates an angle between the shaft of the spinous process and a horizontal line generated by connecting the anterior and posterior articular facets. Angles less than or equal to one degree are termed “horizontal,” and greater than one degree are termed “inclined.”

Shape of pars interarticularis C2: (shallow concave indicates elongation) We define the pars interarticularis as the ridge connecting the anterior and posterior articular facets. The shape of the pars interarticularis in dorsal view is described as shallow-concave or deep-concave. If the distance between the narrowest points of the pars interarticularis is the same size or greater than the width of the dens in dorsal view, we term this “shallow-concave.” If this distance is less than the width of the dens, we call this “deep-concave.”

Shape of cranial bulge C3: (domed indicates elongation) Evaluates the shape of the cranial end of the vertebral body as domed, intermediate, or flat.

Prominence of ventral extension C3: (prominent indicates elongation) We describe the ventral extension as the portion of the cranial bulge that extends distally on the ventral surface of the vertebral body. We evaluate the ratio between the length of the ventral extension compared to the total length of the cranial bulge. When that ratio is greater than 0.5, we term the ventral extension “prominent.” When the ratio is less than or equal to 0.5, we term this “absent.”

Ventral tubercle in relation to cranial bulge C3: (posterior indicates elongation) Evaluates the position of the ventral tubercle in relation to the cranial bulge. The tip of the ventral tubercle can extend anterior to, within, or posterior to the cranial bulge.

Shape of anterior articular facet C3: (oval indicates elongation) Evaluates the shape of the anterior articular facet as round or oval. If the length and width of the facet are approximately equal, this is termed “round,” and if the length is greater than the width, this is termed “oval.”

Connection of anterior articular facet with lamina C3: (disconnected indicates elongation) Evaluates the relationship between the anterior articular facet and the lamina. If the anterior articular facet connects directly with the lamina of the vertebral body, this is termed “connected.” If the anterior articular facet is displaced anteriorly by a longer pillar, this is termed “disconnected.”

Directionality of shaft of ventral tubercle C3: (ventral indicates elongation) Evaluates the orientation of the ventral tubercle as anterior, ventral, or posterior in relation to the vertebral body.

Continuity of anterior arch C3: (interrupted indicates elongation) We define the anterior arch as the ridged connection between the pillar of the anterior articular facet and the ventral tubercle. The pillar is the bony mass that supports the anterior articular facet and connects to the ventral tubercle at the base. If the pillar connects to the ventral tubercle with a ridge, this is termed “uninterrupted.” If the base of the pillar is displaced caudally and there is no ridge connecting the anterior articular facet and ventral tubercle, this is termed “interrupted.”

Height of intertubercular plate C3: (excavated indicates elongation) We define the intertubercular plate as the bony connection between the ventral and dorsal tubercles. We describe its height as tall when greater than 50% of the plate is as high or higher than the spinous process, and excavated when less than 50% of the plate is higher than the spinous process.

Strength of ventral ridge C3: (faint indicates elongation) We define the ventral ridge as the crest extending from the cranial bulge to the caudal end of the vertebral body on the ventral surface. We evaluate this as strong if 50% or more of the ventral ridge is as thick or thicker than the intertubercular plate, and faint if less than 50% of the ridge is as thick as the intertubercular plate.

Highest point of spinous process in relation to foramen transversarium C3 (posterior indicates elongation) Compares the tallest point of the spinous process in lateral view to the position of the foramen transversarium. This can be anterior to, within, or posterior to the foramen transversarium.

Position of spinous process thickness C3 (posterior indicates elongation) Evaluates the position of the thickest concentration of bony material of the spinous process on the dorsal aspect of the vertebra. This can be anterior, central, or posterior.

Presence of laminar ridges C3 (absent indicates elongation) We define the laminar ridges as three crests that radiate from the posterior aspect of the spinous process in dorsal view. These can be present or absent.

Dorsal tubercles in relation to vertebral body C3 (attached indicates elongation) Evaluates the position of the dorsal tubercles in lateral view. If the dorsal tubercles connect directly with the vertebral body, we term this “attached.” If the dorsal tubercles project laterally, we term this “protruding.”

Position of posterior articular facet C3 (posterior indicates elongation) Compares the ventral margin of the posterior articular facet with the caudal end of the vertebral body. The ventral surface of the posterior articular facet can be anterior or posterior to the caudal vertebral body.

Ventral notch C3 (absent indicates elongation) The ventral notch is a skeletal crescent formed between the dorsal tubercle and vertebral body in ventral view. If the dorsal tubercle and ventral body are connected by a plate of bony material, we describe the notch as “absent.”

**Description of Ratios and Percent Posterior**

We devised several calculations to evaluate the slenderness of the vertebrae as well as the proportion of the vertebra posterior to the distal opening of the foramen transversarium (Measurements provided in Supplementary Table 3).

Length to width ratio C2, C3, C7: Calculate the ratio between the maximal length of the cervical vertebra (distance between anterior and posterior articular facets) and the minimal width of the lamina in dorsal view. This ratio puts a numerical value to the slenderness of the vertebra; a higher ratio indicates a vertebra that is longer than it is wide. This ratio encompasses elongation of the vertebral body and lamina, as well as the pillars of the anterior and posterior articular facets.

Percentage of vertebra posterior to foramen transversarium C3: Measure the length of the entire vertebra (distance between anterior and posterior articular facets), and the length of the vertebra posterior to the foramen transversarium (distance between distal opening of foramen and posterior articular facet), and divide the total length by the posterior length. This allows for comparison of caudal vertebral elongation among giraffid species.

**Supplementary Table 1: Table of characters used to measure vertebral elongation**

|  | Pe | Cs | Oj | Gp | Sg | Bm | Sm | Pr | Ba | Gs | Gs |
| --- | --- | --- | --- | --- | --- | --- | --- | --- | --- | --- | --- |
| C2: Height of spinous process | High | High | High | High | High | High | High | / | **Low** | / | **Low** |
| C3: Height of spinous process | **Low** | **Low** | High | **Low** | High | High | **Low** | **Low** | / | **Low** | High |
| C2: Thickness of spinous process | **Thin** | **Thin** | Thick | Thick | Thick | Thick | **Thin** | / | **Thin** | / | **Thin** |
| C2: Verticality of spinous process | **Horizontal** | **Horizontal** | Inclined | / | Inclined | Inclined | Inclined | / | **Horizontal** | / | **Horizontal** |
| C2: Shape of pars interarticularis | **Shallow concave** | **Shallow Concave** | Deep concave | Deep concave | Deep concave | **Shallow concave** | Deep concave | / | **Shallow concave** | / | **Shallow concave** |
| C3: Shape of anterior articular facet | Round | **Oval** | Round | Round | Round | Round | Round | Round | / | **Oval** | **Oval** |
| C3: Directionality of shaft of ventral tubercle | Anterior | **Ventral** | Anterior | Anterior | Posterior | Anterior | Anterior | / | / | **Ventral** | **Ventral** |
| C3: Height of intertubercular plate | Tall | Tall | Tall | Tall | / | Tall | Tall | / | / | **Excavated** | **Excavated** |
| C3: Strength of ventral ridge | Strong | Strong | Strong | Strong | Strong | Strong | Strong | Strong | / | Strong | **Faint** |
| C3: Shape of cranial bulge | Flat | Flat | **Domed** | Intermediate | **Domed** | **Domed** | **Domed** | **Domed** | / | **Domed** | **Domed** |
| C3: Prominence of ventral extension | Absent | **Prominent** | Absent | **Prominent** | Absent | / | **Prominent** | **Prominent** | / | **Prominent** | **Prominent** |
| C3: Orientation of ventral tubercle (in relation to cranial bulge) | Anterior | Within | Within | Within | Within | Within | Within | / | / | **Posterior** | **Posterior** |
| C3: Connection of anterior articular facet with lamina | Connected | Connected | Connected | **Disconnected** | Connected | Connected | **Disconnected** | **Disconnected** | / | **Disconnected** | **Disconnected** |
| C3: Continuity of anterior arch | Uninterrupted | Uninterrupted | Uninterrupted | Uninterrupted | Uninterrupted | Uninterrupted | **Interrupted** | **Interrupted** | / | **Interrupted** | **Interrupted** |
| C3: Highest point of spinous process (in relation to foramen transversarium) | Within | Within | Within | Within | Within | Within | Within | Within | / | **Posterior** | **Posterior** |
| C3: Position of spinous process thickness | Anterior | Central | Central | Central | Central | Anterior | Anterior | Central | / | Central/posterior | **Posterior** |
| C3: Presence of laminar ridges | Strong | Strong | Strong | Strong | **None** | Present | Strong | Strong | / | **None** | **None** |
| C3: Position of dorsal tubercles | Protruding | Protruding | Protruding | Protruding | Protruding | Protruding | Protruding | Protruding | / | Protruding | **Attached** |
| C3: Position of posterior articular facet | Anterior | Anterior | Anterior | Anterior | Anterior | Anterior | Anterior | Anterior | / | Anterior | **Posterior** |
| C3: Presence of ventral notch | Present | Present | Present | Present | Present | Present | Present | Present | / | **Absent** | **Absent** |

Pe: *Prodremotherium elongatum*, Cs: *Canthumeryx sirtensis*, Oj: *Okapia johnstoni*, Gp: *Giraffokeryx punjabiensis*, Sg: *Sivatherium giganteum*, Bm: *Bramatherium megacephalum*, Sm: *Samotherium major*, Pr: *Palaeotragus rouenii*, Ba: *Bohlinia attica*, Gs: *Giraffa sivalensis*, Gc: *Giraffa camelopardalis.* Features used to estimate elongation on the C2 and C3 vertebrae of eleven giraffid species. Items in bold represent taxa exhibiting the elongation state for that given character. The first ten characters (white) measure general vertebral elongation, the next five (blue) measure cranial (anterior-end) vertebral elongation, and the final six characters (green) measure caudal (posterior-end) vertebral elongation.

**Supplementary Table 2: Table of specimens**

| **Taxon** | **Vertebra** | **Specimen** |
| --- | --- | --- |
| *Prodremotherium* | C2 | AMNH 10339a, AMNH 10339b |
| *Prodremotherium* | C3 | AMNH 10339a, AMNH 10339b |
| *Prodremotherium* | C7 | AMNH 10339 |
| *Canthumeryx* | C2 | **NHM** (no number) |
| *Canthumeryx* | C3 | UCB 42020, NMK FT3125 |
| *Canthumeryx* | C7 | UCB42112 |
| *Okapia* | C2 | AMNH 51197, AMNH 51904, AMNH 51218, AMNH 51222, AMNH 51198 |
| *Okapia* | C3 | AMNH 51197, AMNH 51904, AMNH 51218, AMNH 51222, AMNH 51198, AMNH 51213, AMNH 51214, AMNH 113802, AMNH 51223 |
| *Okapia* | C7 | AMNH 51197, AMNH 51904 |
| *Giraffokeryx* | C2 | AMNH 19527, NMK FT3079, AMNH 19672, NMK 74.64 |
| *Giraffokeryx* | C3 | NMK FT2010.62, AMNH 29987 |
| *Sivatherium* | C2 | NHM 39528 |
| *Sivatherium* | C3 | NHM 16225A, NHM 1129, NHM 074 |
| *Sivatherium* | C7 | NHM 18289 |
| *Bramatherium* | C2 | YPM 13881 |
| *Bramatherium* | C3 | YPM 13881 |
| *Samotherium* | C2 | PIM 430, NHM 4250(292) |
| *Samotherium* | C3 | PIM 1002, PIM 432, PIM 1011, PIM 1012, PIM 431, NHM 4250 (291), NHM 4247 (299) |
| *Samotherium* | C7 | PIM 436 |
| *Palaeotragus* | C3 | **PIUW** (no number), PIU M1177 |
| *Bohlinia* | C2 | PLA 3308/91, PLA 3307/91, **RMS** (no number) |
| *Bohlinia* | C7 | PLA 3515/91 |
| *G. sivalensis* | C3 | NHM 39747 |
| *G. camelopardalis* | C2 | AMNH 82001, AMNH 27666, AMNH 5343, AMNH 27752 |
| *G. camelopardalis* | C3 | AMNH 82001, AMNH 27666, AMNH 5343, AMNH 27752 |
| *G. camelopardalis* | C7 | AMNH 82001, AMNH 27666, AMNH 5343 |

This lists the specimens utilized in this study, with the vertebral number and museum number. AMNH- American Museum of Natural History, New York; NHM- Natural History Museum, London; NMK- National Museums of Kenya, Nairobi; PIM- Paleontological Institute Münster; PIU- Paleontological Institute of Uppsala; PIUW- Paleontological Institute Vienna; PLA- Paleontological Laboratory Athens; RMS- Riksmuseum of Natural History, Stockholm; UCB- Museum of Paleontology University of California Berkley; YPM- Yale Peabody Museum, New Haven

**Supplementary Table 3: Table of ratios and percentage of vertebrae posterior to the foramen transversarium**

|  | **C2 L:W Ratio range (n)** | **C3 L:W Ratio range (n)** | **C7 L:W Ratio range (n)** | **Percentage range of C3 vertebra posterior to the foramen transversarium** |
| --- | --- | --- | --- | --- |
| *Prodremotherium* | 3.72-3.74 (2) | 1.8-2.11 (2) | 1.19 (1) | 20.1-27.5 |
| *Canthumeryx* | 3.123 (1) | 2.28-3.24 (2) | 1.54 (1) | 39 |
| *Okapia* | 3.67-5.43 (3) | 1.41-2.07 (9) | 1.38 (2) | 35.5-39 |
| *Giraffokeryx* | 4.59 (1) | 1.36-2.21 (3) | / | 18.6-43.2 |
| *Sivatherium* | 4.57 (1) | 1.2 (1) | 1.06 (1) | 37 |
| *Bramatherium* | 2.24 (1) | 1.3 (1) | / | 25.1 |
| *Samotherium* | 4.2 (1) | 2.12-3.36 (6) | 1.82 (1) | 25.4-37.3 |
| *Palaeotragus* | / | 2.81-3.5 (2) | / | 32.8-38.6 |
| *Bohlinia* | 5.52-5.85 (2) | / | 2 (1) | / |
| *G. sivalensis* | / | 6.63 (1) | / | 39 |
| *G. camelopardalis* | 9.18-10.64 (4) | 8.9-10.8 (4) | 3-3.75 (3) | 49.8-56.3 |

Range of length to width ratios of C2, C3, and C7 vertebrae, as well as the percentage of the C3 vertebrae posterior to the caudal opening of the foramen transversarium of all giraffid species studied. The length to width ratios were calculated by dividing the maximal length of the cervical vertebra (distance between anterior and posterior articular facets) by the minimal width of the lamina in dorsal view. *Bohlinia attica, Giraffa sivalensis,* and *Giraffa camelopardalis* exhibit the largest ratios, with the modern giraffe exhibiting the most slender vertebrae. The modern giraffe also demonstrates the largest percentage of vertebrae as posterior to the foramen transversarium.
